# Anti-N-Methyl-D-Aspartate Receptor (Anti-NMDAR) Encephalitis in a Young Pregnant Woman With a 10-Year Follow-Up: A Case Report

**DOI:** 10.7759/cureus.70587

**Published:** 2024-10-01

**Authors:** Rwan Almalki, Amal Almohawes, Reef Alajaji, Hesham Eissa

**Affiliations:** 1 Adult Neurology, King Fahad Military Medical Complex, Dhahran, SAU; 2 Adult Neurology, Zayed Military Hospital, Abu Dhabi, ARE

**Keywords:** anti-n-methyl-d-aspartate receptor, autoimmune, case report, encephalitis, pregnancy

## Abstract

Anti-N-methyl-D-aspartate receptor (anti-NMDAR) encephalitis is an autoimmune disease of the central nervous system with unknown etiology and multiple triggers. It is clinically characterized by the onset of psychiatric symptoms, seizures, memory disturbance, and cognitive decline. We present a young, newly married woman in the first trimester of pregnancy who presented with purely psychiatric manifestations, fever, and two instances of generalized tonic-clonic seizures. She eventually progressed to develop a decreased level of consciousness and hemodynamic instability. A diagnosis was made of anti-NMDA encephalitis. Her symptoms progressed even after the administration of two intravenous immunoglobulin (IVIG) trials, steroids, and three anti-seizure medications until she experienced a spontaneous abortion. She then gradually returned to her normal baseline condition. This case underscores the importance of suspecting anti-NMDAR encephalitis in pregnant patients with acute onset of psychiatric manifestations. Anti-NMDAR encephalitis can be a difficult, challenging, and exhausting diagnosis for both the patient and treating physicians, particularly in pregnant patients where diagnostic and therapeutic options may be limited due to concerns for fetal safety. However, this case provides evidence that anti-NMDAR encephalitis during pregnancy can have a good prognosis.

## Introduction

Anti-N-Methyl-D-Aspartate Receptor (anti-NMDAR) encephalitis is a newly recognised autoimmune disease of the central nervous system. It was first described in 2007 as a paraneoplastic condition [1]. Since then, the number of reported cases of anti-NMDAR encephalitis, whether accompanied by tumours or not, has rapidly increased. Anti-NMDAR encephalitis is the most common autoimmune encephalitis and is most prevalent in young females with a median age of 21 years [1]. The aetiology of anti-NMDAR encephalitis is still unclear; however, it is reportedly provoked by neoplasia and paraneoplastic syndromes, especially NMDAR-expressing ovarian teratomas, and it has been found to occur secondary to viral infections such as herpes simplex virus, enterovirus, and West Nile virus (WNV), bacterial infections such as listeria, streptococcus, tuberculosis, and fungal infections in immunocompromised patients [2-4].

There are limited reports of anti-NMDAR encephalitis occurring during pregnancy, and the significance of this condition in pregnancy lies in the limited data and clinical guidelines available to guide treatment decisions [5]. This knowledge gap makes it difficult to balance effective maternal treatment with minimizing risks to the fetus, underscoring the need for more research and clinical reports to optimize management strategies. Thus, this case report provides valuable insights into the clinical course, management decisions, and long-term outcomes of pregnant patients with anti-NMDAR encephalitis, contributing to the growing body of evidence needed to refine treatment protocols and improve prognosis for both mother and child.

## Case presentation

The patient was a 22-year-old pregnant Saudi woman (gestational age: 16 weeks and five days) with no known medical or surgical history. The patient’s family brought her to the emergency department (ED) at various healthcare facilities multiple times over a two-week period due to acute onset of psychiatric symptoms in the form of agitation, instances of aggression, bizarre behaviour, and apathy, and two brief attacks of generalized tonic-clonic seizures with no clear diagnosis or management provided, in addition to documented fever events over the two days prior to ED presentation. The patient was finally referred to the neurology department as a suspected case of epilepsia partialis continua (EPC) with continued right upper limb jerky movement on September 10, 2013.

The neurology team’s initial assessment established that the patient was a young Saudi woman who was underweight, febrile (38.8°C) and had tachycardia (heart rate of 117 beats/minute), a blood pressure of 160/80 mmHg. She looked confused, and when she opened her eyes, she was not able to focus her vision. Her pupils were bilaterally rounded, 2 mm in diameter, and had a sluggish response to light. She had neck stiffness with a positive Brudzinski sign and a continuous right upper limb jerky movement. In addition, she had decreased deep tendon reflexes all over her body and a downward flexor plantar response. After cessation of the involuntary movement in ED, the patient was shifted to the intensive care unit (ICU) under the care of Neurology.

**Figure 1:**
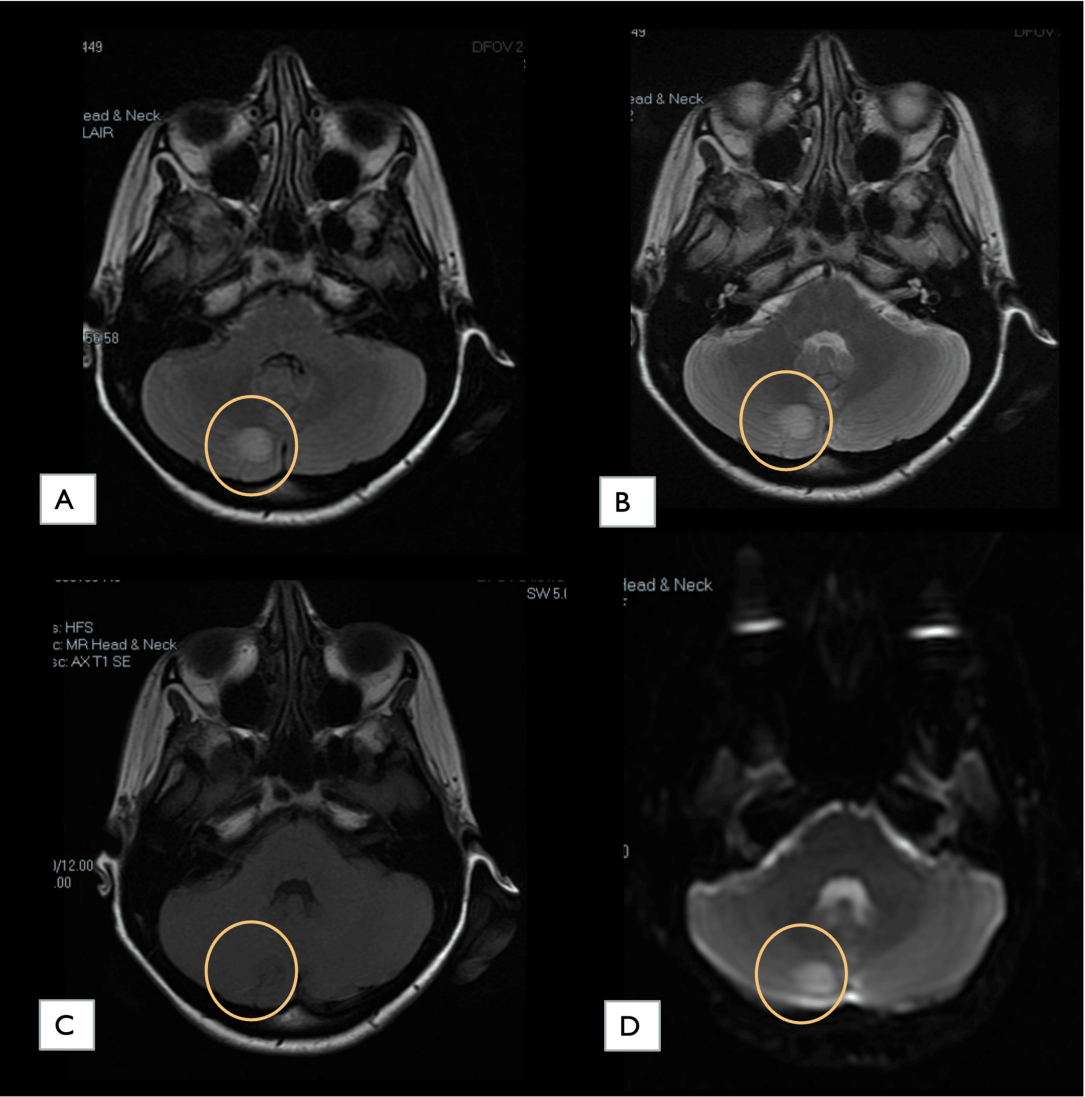
Brain MRI images showing an altered signal intensity along the posterior aspect of the right cerebellar hemisphere. In FLAIR (A) and T2-weighted (B) images, it appeared as a localized hyperintensity. In T1-weighted images, it appeared as subtle hypointensity (C). Mild signal restriction was observed in the DWI sequence (D). Considering the patient’s clinical results, encephalitis was determined to be the top primary diagnosis. FLAIR: fluid-attenuated inversion recovery; DWI: diffusion-weighted imaging

A few hours later, she started to have right upper limb focal clonic motor seizures that evolved to generalised tonic-clonic. We then initiated an empirical treatment regimen for a suspected case of meningoencephalitis that included the administration of daily 2 g IV ceftriaxone, 500 mg IV acyclovir every eight hours, 750 mg IV vancomycin every 12 hours, and 1 g IV pulse steroid, in addition to 500 mg levetiracetam every 12 hours and 100 mg IV phenytoin every eight hours. The obstetrics and gynaecology (OB-GYN) team was involved to determine the viability of the pregnancy. Their assessment showed a single viable fetus, which was within the normal ranges for fetal biometry for gestational age. After ensuring fetal status, we arranged for an urgent magnetic resonance imaging (MRI) of the brain without contrast. As shown in Figure 1, the resultant images showed an area of altered signal intensity along the posterior aspect of the right cerebellar hemisphere that supported the diagnosis of meningoencephalitis.

On the second day of admission, the neurology team performed a lumbar puncture to obtain cerebrospinal fluid (CSF) for analysis. The results are shown in Table 1. Serum serology tests revealed negative results (Table 2). 

**Table 1:** CSF analysis CSF: cerebrospinal fluid

Parameters	Value	Refrances
CSF Colour	Clear colourless fluid	Clear
White Blood Cell (WBC)	4 cells/mm3	<5 cells/mm3
Blood Cell (RBC)	6 cells/mm3	<1 cells/mm3
CSF Protein	22 mg/dL	(15 – 60) mg/dL
CSF Glucose	5.9 mmol/L	2/3 serum glucose
Serum Glucose	5.7 mmol/l	(4.4–7) mmol/l
Gram stain and culture	Negative	Negative

**Table 2:** Serology test results

Serology	Findings
Tuberculosis	Negative
Brucellosis	Negative
Hepatitis	Negative
Herpes simplex	Negative
Cytomegalovirus	Negative
Epstein-Barr virus	Negative
Human Immunodeficiency Virus	Negative
Malaria	Negative
Toxoplasmosis immunoglobulin (IgM)	Negative
Typhoid	Negative
Parvovirus B19	Negative

**Figure 2:**
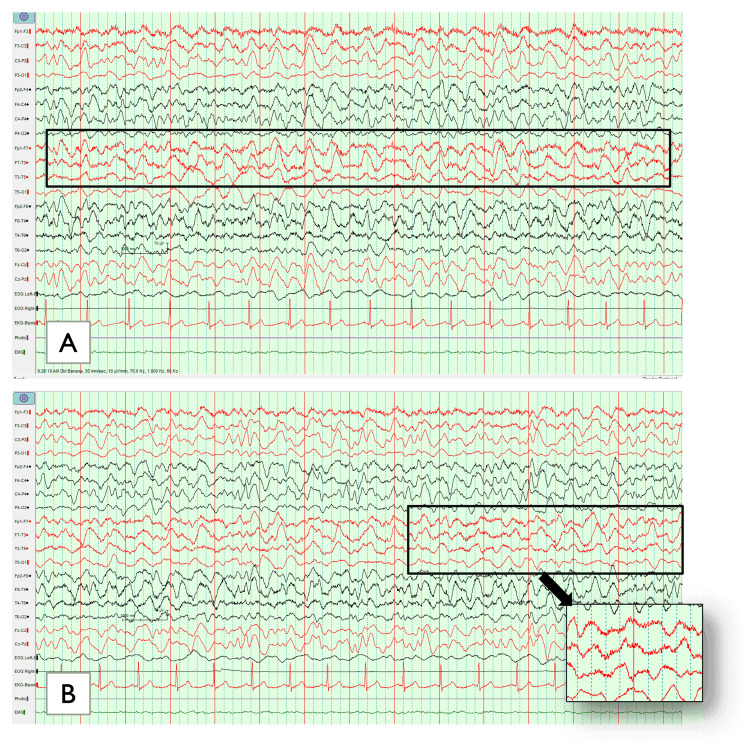
Delta brush is a novel ictal EEG pattern in anti-NMDAR encephalitis. (A) It is characterized as fast-spiking activity on background of generalized delta rhythm; (B) Showing the detailed characteristics of delta activity at 1-3 Hz with superimposed bursts of rhythmic 12-30 Hz activity NMDAR: N-methyl-D-aspartate receptor

In addition, negative results were obtained for polymerase chain reaction (PCR), thyroid function test, and antinuclear antibody (ANA) test. This narrowed the differential diagnosis list to metabolic versus autoimmune encephalopathy. Therefore, ceftriaxone and vancomycin were discontinued. A second lumbar puncture was attempted without success. With the onset of new athetoid movement in the right upper limb, an electroencephalogram (EEG) was conducted. The results showed a generalized delta brush with recurrent right frontal prominent fast spike activity (Figure 2).

One week later, the patient started to have generalized tonic-clonic seizures and fluctuating levels of consciousness. Another EEG was conducted after the levetiracetam dose increased to 1g twice a day, and the results revealed a generalized slowing, which was suggestive of encephalopathy. After multiple unsuccessful lumbar puncture attempts, the anaesthesia team was involved; however, the results were inconclusive and implied that the puncture was traumatic in nature. Consequently, the patient continued to be managed as a case of partially treated bacterial meningitis, and antibiotics were resumed. With no noticeable clinical improvement and multiple daily generalized tonic-clonic seizures, an MRI with gadolinium was planned, and the risks and benefits were thoroughly explained to the patient’s family. The results of the brain MRI indicated that the disease was stationary, with no significant radiological changes. After the repeated MRI, the patient suddenly developed a new stridor and episodes of tachycardia, and her O2 saturation level dropped to 85% on face mask. Therefore, she was sedated and intubated.

On day 10 of admission, with the patient displaying no signs of minor improvement, very high levels of antibodies specific for NMDAR were detected in the patient’s serum and CSF (titer: 1.32). She was subsequently treated with 1 g of IV methylprednisolone and IVIG (0.4 mg/kg/day) for five days. When the IVIG course was completed, the outcome was again disappointing, with the patient exhibiting insignificant progress. A multidisciplinary team meeting was held to discuss further workups and prognosis. The team planned to perform a tracheostomy, another lumbar puncture, and an abdominal/pelvic MRI to rule out teratoma as soon as the patient’s general condition allowed. After a failed trial to wean the patient off ventilatory support, the patient was re-intubated and underwent a tracheostomy on day 20 after admission.

A repeat lumbar puncture was successfully performed, and the CSF analysis results were comparable to those of the initial CSF analysis (Table 3).

**Table 3:** Repeat CSF analysis CSF: cerebrospinal fluid; NMDAR: N-methyl-D-aspartate receptor

Parameters	Value	Refrances
CSF Colour	Clear colourless fluid	clear
White Blood Cell (WBC)	2 cells/mm3	<5 cells/mm3
Blood Cell (RBC)	28 cells/mm3	<1 cells/mm3
CSF Protien	21 mg/dL	(15 – 45) mg/dL
CSF Glucose	5.3 mmol/L	2/3 serum glucose
Serum Glucose	5.3 mmol/l	(4.4–7) mmol/l
Gram stain and culture	Negative	Negative
Anti-NMDAR antibody titre	1.40	(0-0.5)

As a result, a second five-day course of IVIG was started; however, there was no noticeable improvement. The patient's neurological status showed a spontaneous eye opening and poor eye fixation, as well as recurrent focal seizures in the form of jerky movements of the right upper limb and facial twitches. Phenobarbital (50 mg, intravenous, three times a day) was added to the patient’s existing antiseizure medications, which were levetiracetam (1 gram, twice a day) and midazolam (10 mg/hour, infusion). Despite the patient receiving three antiseizure medications, she continued to have daily focal seizures.

**Figure 3:**
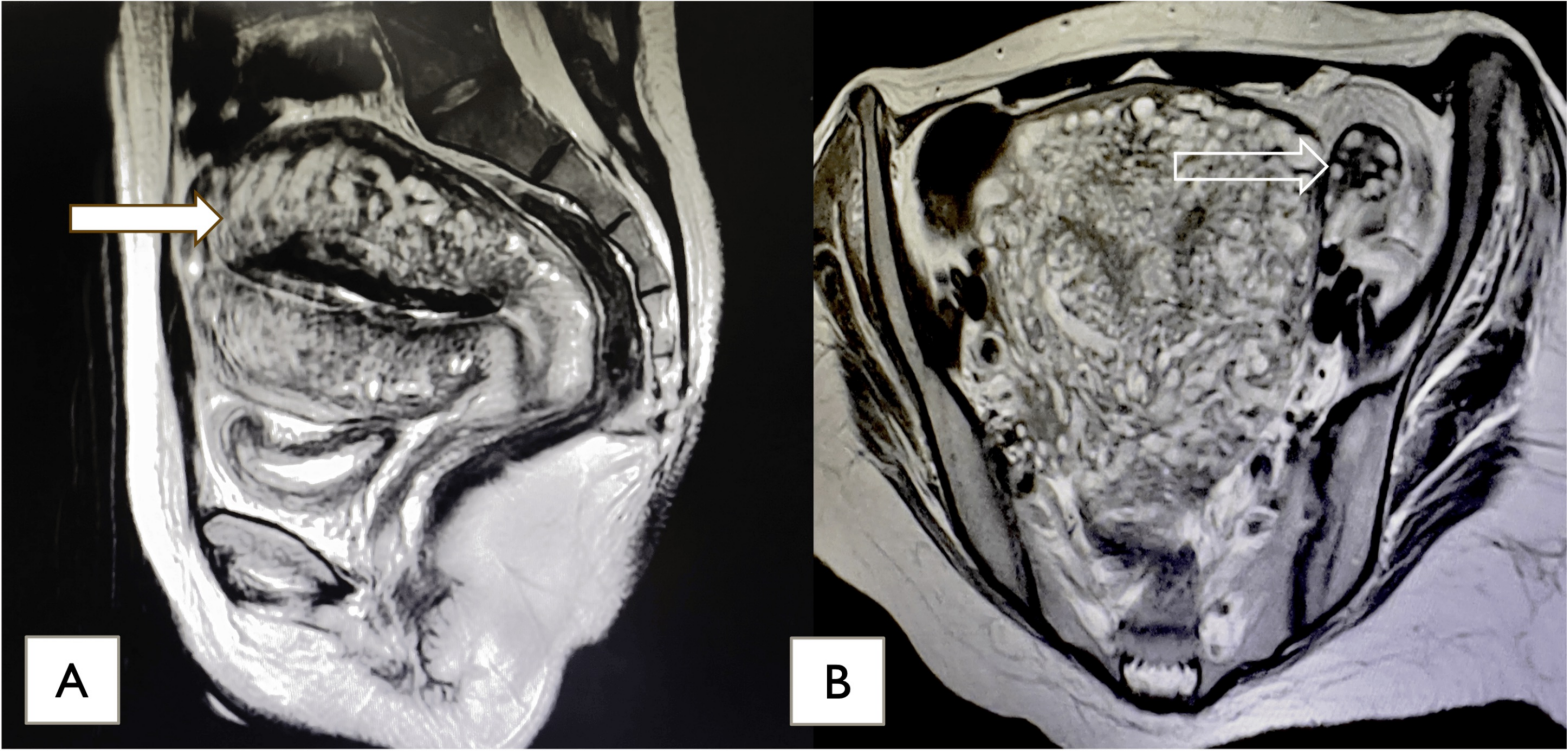
Pelvic MRI: (A) Sagittal view showing large hypervascularized uterus (post partum) marked by the arrow; (B) Axial view showing left ovary measuring 1.8x1.1x2.3 cm with multiple peripheral follicles (hollow arrow) and right ovary measuring 1.4x2.6x1.6 cm normal signal intensity and multiple peripheral relocated follicles. No adnexal mass could be seen.

On day 45 of admission, the patient started to develop vaginal spotting. She was assessed by the OB-GYN team, who reported that she was undergoing an abortion at 21+ weeks of gestation. The nursing team was instructed to observe for spontaneous abortion. The following morning, while being assessed by the morning shift nurse, the patient was found to have experienced a spontaneous abortion. A baby boy was found between her thighs while she was clinically and vitally stable. Pelvic MRI showed normal findings, ruling out any possibility of a teratoma (Figure 3). A series of dramatic clinical improvements occurred after the abortion event. The patient started to move all four of her extremities, focus her vision, and pull on the nasogastric tube and intravenous access. One week after the abortion, the patient was removed from sedation. CSF analysis was repeated, and the sample showed a high titer of anti-NMDAR antibodies 1:32, (reference value 0-0.5). At this point, the patient was in a sufficiently stable condition to proceed with abdominal and chest CT scans, which showed normal findings with mild hepatomegaly and no focal lesions. 

Two weeks later, she was clinically improved and ready to be transferred to a ward setting. The patient’s communication skills had markedly improved from vocalization and head nodding to speaking clear words and the dosage of dexamethasone was switched to oral prednisolone (20 mg daily). After undergoing aggressive physiotherapy, speech therapy, and rehabilitation sessions for one month, the patient was able to speak clearly and walk with support. At the time of discharge (December 26, 2013), she had been admitted for 18 weeks and had been seizure-free for more than 45 days. At discharge, her medication consisted of levetiracetam (750 mg twice a day), phenytoin (100 mg three times a day), and prednisolone (tapering plan).

**Figure 4:**
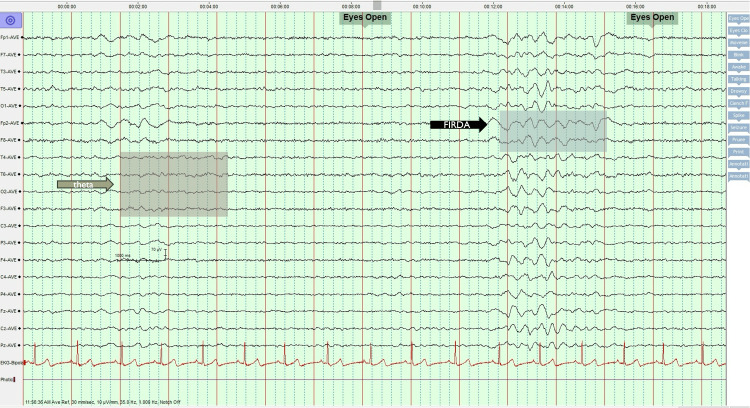
Background of 5 Hz theta activity, intermingled with slower and larger amplitude 3 Hz delta activity sometimes generalized (delta brush). Intermittent and rhythmic 3 Hz delta activity maximum frontally; Frontal intermittent rhythmic delta activity (FIRDA). No epileptiform discharges seen.

Again, In 2017, the patient presented to our emergency department at 13 weeks’ gestation with a complaint of headache and a single generalized tonic-clonic seizure at home. At that time, she was not on antiseizure medications (stopped by herself). The neurology team admitted her for 24 hours of observation, and levetiracetam (500 mg twice a day) was resumed. An EEG was performed and showed a background of 5 Hz theta activity intermingled with slower and larger amplitude 3 Hz delta activity at times (Figure 4). 

**Figure 5:**
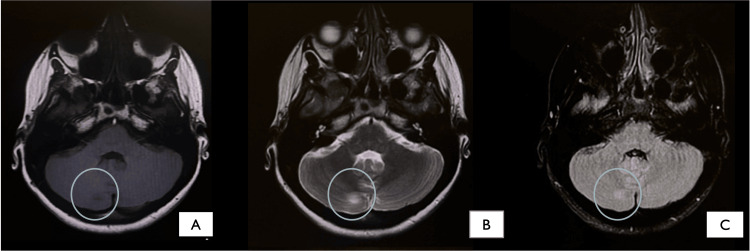
Regression of right cerebellar lesion seen on sequences: (A) T1, (B) T2, (C) FLAIR FLAIR: fluid-attenuated inversion recovery

Brain MRI was also performed, and the results showed regression of the previously reported right cerebellar encephalitis, with no other abnormal findings as shown in Figure 5.

No serum anti-NMDAR antibodies were detected. Thus, the patient was discharged and followed regularly throughout her pregnancy. No breakthrough seizures were reported. She safely delivered her baby with no pre- or postpartum complications. After delivery, the patient was seen annually in the neurology clinic, and no seizures were reported between 2017 and 2021. She was last seen in the clinic in March 2021, at which time she stopped taking levetiracetam by herself, and was subsequently lost to follow-ups. On December 29, 2023, the patient attended a consultation before her third delivery.

She has been in remission for more than seven years now. Hence, after being diagnosed with anti-NMDAR encephalitis, the patient uneventfully delivered two babies with no complications or recurrence of previous symptoms.

## Discussion

Anti-NMDAR encephalitis is the most common type of encephalitis reported during pregnancy. The pathophysiology of this condition involves antibodies (IgG) targeting the NMDAR NR1 subunit in the central nervous system, which in turn leads to internalization of the receptors (which are glutamate receptors), a reduction in the neuronal calcium influx, and a decrease in receptor-dependent synaptic activity [2].

Anti-NMDAR encephalitis has a wide variety of symptoms, including psychiatric disorders, epilepsy, speech disorders, motor weakness, memory impairment, autonomic dysfunction, and changes in the level of consciousness [2,6]. Psychiatric symptoms are usually the first alarming and noticeable findings, especially in patients without a previous psychiatric background, as in our case [7]. In addition, patients with anti-NMDAR encephalitis may experience fatigue, difficulty concentrating, cognitive challenges, incoordination, memory disturbance, lack of motivation, personality change, irritability, or depression [8,9].

Patients with encephalitis are 16 times more likely to develop seizures than the general population, and in the first two years after an anti-NMDAR encephalitis attack, patients are more likely to develop seizures than those with other types of autoimmune encephalitis. Furthermore, the risk of late onset of unprovoked seizures for patients who developed seizures during the acute attack is 10% at five years and rises to 22% at 20 years [10].

Due to the potential adverse effects of diagnostic and therapeutic interventions on the fetus, managing anti-NMDAR encephalitis during pregnancy presents unique challenges. While diagnosing anti-NMDAR encephalitis is heavily reliant on clinical findings, supportive radiological findings are crucial when identifying differential diagnoses. However, it has been reported that there is a risk of neonatal death and stillbirth associated with exposure to MRI with gadolinium at any time during pregnancy [11]. The risk of major congenital anomalies associated with the use of first-generation antiseizure medications such as phenytoin, phenobarbital, and valproate is another serious challenge faced when treating pregnant patients.

Immunotherapy is the first-line treatment and a cornerstone in the management. Given that pregnancy was the primary challenge in our case, it significantly influenced our management approach and treatment decisions. Corticosteroid therapy is considered safe during pregnancy, with a generally low risk of major congenital malformations (MCMs). However, its use may be associated with complications such as premature rupture of membranes and low birth weight. Some studies have also linked corticosteroids to an elevated risk of preterm delivery, intrauterine growth restriction, and small for gestational age. Although the available information regarding the use of plasmapheresis (PLEX) in pregnant patients is limited, established guidelines endorse the use of PLEX as an alternative form of treatment when administered through a well-organized multidisciplinary team.

In our case, the decision was made to combine steroids and IVIG. The safety profile of IVIG administration during pregnancy has been supported by several observational studies. Fetal exposure to IVIG is not associated with an increased risk of MCMs or fetal distress. Furthermore, IVIG may provide a protective effect on the fetus by blocking the transplacental transfer of autoimmune encephalitis (AE)-related immunoglobulin G (IgG) antibodies. Other second-line options such as rituximab (RTX), cyclophosphamide (CYC), and azathioprine (AZA) were only administered in a minority of reports [12].

Overall, most patients with anti-NMDAR encephalitis have a good clinical prognosis. However, in some patients, it results in intractable epilepsy, severe mental disorders, or even death [9]. One study reported that epileptic attacks slowly and gradually disappeared and that involuntary movement and higher neurological function disturbance occurred within a period of 1-12 months. The mortality rate of anti-NMDAR encephalitis can reach 10% [13].

The patient in our case was a healthy, young, pregnant woman who presented with pure psychiatric manifestations and fever at 16 weeks of gestation. She also experienced an altered mental status, seizures, episodes of dysautonomia, and O2 desaturation that mandated mechanical ventilation. For two months post admission, despite our efforts to assess and treat the patient by performing various workups and imaging protocols, continually adjusting her antiseizure and antibiotic regimes, and administering two IVIG trials, the patient showed no appreciable response. However, she returned to her normal baseline a few weeks after experiencing a spontaneous abortion. Notably, PLEX service was not available in our center, and rituximab, an effective second-line immunotherapeutic agent, was not administered to this patient due to insufficient data available on its safe use during pregnancy when our patient initially presented in 2013.

The recovery phase of anti-NMDAR encephalitis can be up to 18 months in duration. Relapse after resolution is commonly encountered and typically seen within the first two years; the prevalence is 12% [14].

## Conclusions

This case underscores the complex and challenging nature of diagnosing and managing anti-NMDAR encephalitis during pregnancy, highlighting the delicate balance between treating the mother effectively and ensuring fetal safety. The clinical presentation of anti-NMDAR encephalitis often includes early psychiatric symptoms, which may be overlooked or misdiagnosed, particularly in pregnant patients where such symptoms could be attributed to hormonal or psychological changes. Our patient presented with predominantly psychiatric symptoms and fever, later developing seizures, autonomic instability, and respiratory compromise, which necessitated intensive care management and mechanical ventilation. The absence of response to first-line therapies such as corticosteroids and IVIG, until after a spontaneous abortion further illustrates the potential impact of pregnancy on the disease course and treatment efficacy. The lack of established guidelines on the safety and effectiveness of second-line therapies, such as plasmapheresis and rituximab, complicates treatment decisions in pregnant patients. In our case, the inability to use plasmapheresis and the decision to withhold rituximab due to limited safety data at the time significantly influenced the management approach.

This case emphasizes the need for a multidisciplinary approach and close monitoring of both mother and fetus, along with continuous re-evaluation of treatment strategies. Long-term follow-up is essential given the risk of relapse, which is most common within the first two years post recovery. Our experience also underscores the importance of building a robust evidence base through case reports and clinical studies to guide management in pregnant patients with anti-NMDAR encephalitis. Ultimately, increased awareness, early recognition, and prompt initiation of immunotherapy are key to improving outcomes. The case demonstrates that a favourable prognosis is achievable, even in severe and complex presentations, provided that clinicians remain vigilant and flexible in adapting treatment strategies to address the unique challenges posed by pregnancy. Further research is needed to develop standardized treatment protocols and to establish the safety and efficacy of second-line therapies for anti-NMDAR encephalitis in pregnant patients.
